# REC8 suppresses tumor angiogenesis by inhibition of NF-κB-mediated vascular endothelial growth factor expression in gastric cancer cells

**DOI:** 10.1186/s40659-020-00307-1

**Published:** 2020-09-21

**Authors:** Miao Liu, Wanfu Xu, Mingmin Su, Pingsheng Fan

**Affiliations:** 1 Anhui Provincial Hospital, Cheeloo College of Medicine, Shandong University, Jinan, 250021 Shandong China; 2 The First Affiliated Hospital of USTC, Division of Life Sciences and Medicine, University of Science and Technology of China, Heifei, 230031 Anhui China; 3 Department of Gastroenterology, Guangzhou Women and Children’s Medical Center, Guangzhou Medical University, Guangzhou, 510623 China; 4 Guangzhou Institute of Pediatrics, Guangzhou Women and Children’s Medical Center, Guangzhou Medical University, Guangzhou, 510623 China; 5 Department of Cancer Biology and Therapeutics, School of Pharmacy and Pharmaceutical Sciences, Cardiff University, Wales, CF103AT UK; 6 Department of Oncology, Anhui Provincial Cancer Hospital, Hefei, Anhui 230001 P.R. China

**Keywords:** REC8, Tumor angiogenesis, VEGF, Gastric cancer

## Abstract

**Background:**

Tumor angiogenesis is an essential event for tumor growth and metastasis. It has been showed that REC8, a component of the meiotic cohesion complex, played a vital role in Epithelial-Mesenchymal Transition (EMT) in gastric cancer. However, the role of REC8 in gastric cancer angiogenesis remains to be identified.

**Results:**

Inhibition of REC8 expression in gastric cancer cells contributed to tumor angiogenesis in the gastric cancer microenvironment. The clinical analysis demonstrated that the loss of REC8 in gastric cancer with enrichment of MVD. Depletion of REC8 expression in gastric cancer cells significantly increased tube formation of human umbilical vein endothelial cells (HUVECs), which is attributed to enhancement of vascular endothelial growth factor (VEGF) secretion caused by REC8 slicing. While addition of neutralizing antibody targeted VEGF into supernatant drastically reversed the effect of REC8 loss in gastric cancer cells on tube formation. Mechanistic analyses indicated that ablation of REC8 promotes nuclear factor-κB (NF-κB) p65 activity and its downstream gene *VEGF* expression, leading to tube formation.

**Conclusions:**

These results demonstrated a novel REC8 function that suppressed tumor angiogenesis and progression by attenuation of VEGF in gastric cancer microenvironment.

**Electronic supplementary material:**

The online version of this article (10.1186/s40659-020-00307-1) contains supplementary material, which is available to authorized users.

## Background

Despite tremendous effort has been made to improve the effectiveness of treatment, gastric cancer is a common type of malignant tumor with relatively poor prognosis and presents a serious threat to global health [1], ranking fifth in terms of incidence and third in terms of mortality worldwide [2]. A growing body of evidences indicated that angiogenesis, one of the cancer hallmarks, is key requirement for tumor growth, invasion [3]. Targeting vessel formation and disturbing tumor vasculature have been among the primary strategies in cancer treatment [4]. However, the contribution of tumor angiogenesis remained elusive.

Angiogenesis is defined as the formation of new blood vessels from preexisting vessels and has been characterized as an essential process for tumor cell proliferation and viability [5], while a large number of evidences indicated that VEGF is widely accepted as a primary inducer of angiogenesis [6, 7]. VEGF, binding to its receptors, especially VEGFR2, appeared to be a key factor in pathological situations that involved in tumor neovascularization [8]. Evidence has been shown that inhibitors of VEGF and VEGFR2 reduced endothelial cell proliferation, migration and survival that led to regression of vessel density and decrease vascular permeability, thereby slowing tumor growth [9, 10]. however, the upstream of these transcription factors remained to be fully elucidated.

REC8, a key meiosis-specific component of the cohesion complex, has been implicated in DNA damage repair and maintenance of chromosome stability [11, 12]. It has been reported that REC8 was hypermethylated in melanomas and malignant gastrointestinal stromal tumors [13–15], suggesting the low level of REC8 expression in tumor and a potential tumor suppressor. These findings suggested that the multifaceted REC8-mediated anticancer effects played a causal but unclear role in mammalian oncogenesis. However, there is no available reports about the function of REC8 on tumor angiogenesis. In this study, we are for the first time to demonstrate that REC8 played an anti-angiogenic role in the tumor angiogenesis in gastric cancer and defined a novel model in which REC8 inhibited NF-κB pathway in gastric cancer cells through suppression of VEGF expression, leading to inhibit angiogenesis. Most importantly, the expression of REC8 level is negatively correlation with MVD in gastric cancer. This finding not only provided further support for the tumor suppressor role of REC8, but also added a novel link between abnormal cell meiosis and tumor angiogenesis.

## Materials and methods

### Chemicals and reagents

The simpleChIP® plus enzymatic chromatin IP Kit was from Cell Signal Technology (Danvers, MA, USA). All-in-One First-Strand cDNA Synthesis Kit and All-in-One qPCR Mix were from GeneCopoeia (Rockville, MD, USA). Antibodies used for immunoblotting and IP assays were as follows: the NF-KB p65 primary antibody for chromatin IP was purchased from CST; primary antibodies against VEGF were from Abclonal (Cat: A17877, Wuhan, China);

### Cell lines, cell cultures and transfection

The gastric cancer cell lines of poor differentiation, BGC823 and AGS-1, human umbilical vein endothelial cells (HUVECs) were obtained from Cell Bank of the Chinese Academy of Sciences (Chinese Academy of Sciences, Shanghai, China) and cultured in Dulbecco’s Modified Eagle’s Medium (DMEM, Gibco) supplemented with 10% (v/v) fetal bovine serum (FBS) at 37 °C in a humidified atmosphere of 5% CO_2_. The plasmids were transfected into cells with Hilymax according to manufacturer’s protocol.

### Generation of stable cell line

The lentivirus control shRNA (shCTL: sc-108080) and REC8 shRNA (shREC8: sc-106878-V) were purchased from Santacruz Biotechnology, INC. Puromycin was purchased from sigma and used to select for stably infected cells.

### RNA extraction and quantitative real-time PCR

Total RNA was extracted from cultured cells with trizol reagent (Invitrogen, Carlsbad, CA, USA) according to the manufacturer’s protocol. All cDNA samples were prepared using an All-in-one First-stand cDNA synthesis kit (GeneCopoeia, MD, USA). Quantitative real-time PCR (RT-qPCR) analyses were performed with an all-in-one qPCR mix (GeneCopoeia) according to manufacturer’s instructions using an ABI StepOne-Plus™ qPCR system. The primer for VEGF: Forward: 5′-TGCAGATTATGCGGATCAAACC-3′; Reverse: 5′-TGCATTCACATTTGTTGTGCTGTAG-3′; REC8: Forward: 5′-CATCCCACCAGAAGAACGG-3′; Reverse: 5′-GCACCAAAGGCATCTCCAT-3′; β-actin: Forward 5′-ATCGTGCGTGACATTAAGGAGAAG-3′; reverse:5′-AGGAAGGAAGGCTGGAAGAGTG-3’.

### Endothelial cell tube formation assay

As described in Xu et al*.* study [16], 96-well plates were coated with matrigel basement membrane matrix (BD Biosciences) and then allowed to polymerize at 37 °C for at least 30 min. HUVECs were treated with conditional medium for 6–8 h, tubes formation of HUVECs can be visualized and the number of nodes (defined as when at least three cells formed a single point) per image was quantified as described [17].

### Immunoblotting assays

As described in the study [18], cell lysates were lysed with 2 × loading sample buffer and analyzed by immunoblotting to detect proteins. Briefly, the protein was transferred to a 0.22 μm nitrocellulose transfer membrane. The membrane was blocked with 5% (w/v) milk in PBS/ 0.05% (v/v) Tween-20 and then incubated with the indicated antibody overnight. This was followed by incubation with a horseradish peroxidase secondary antibody (Jackson ImmunoResearch) for 1 h at room temperature. Proteins were detected using enhanced chemiluminescence substrates (Perkin Elmer). The antibodies listed as followed: REC8(abgent, AP13570c,1:2000 for WB; proteintech 11913-1-AP, 1:200 for IP), VEGF(abclonal, A12303,1:2000 for WB; MAB293, 1:50 for neutralize), NF-κB p65 (abclonal, A11202, 1:2000 for WB), Phospho-NF-κB p65(Ser536) (Ser536) (abclonal, AP0124, 1:2000 for WB). α-tubulin (abclonal, AC012); β-actin (abclonal, AC004).

### Enzyme-linked immunosorbent assays (ELISA)

Quantitative measurement of VEGF secreted into conditional medium was determined using ELISA according to the manufacturer’s protocol (elabscience, E-EL-H0111c).

### Immunohistochemistry (IHC) of human tissue microarrays

A human gastric cancer tissue microarray was purchased from Alenabio. Gastric cancer samples were immunostained against indicated antibodies as previously described [18]. Briefly, the slides were dewaxed and rehydrated in distilled water, sections were immersed in citrate buffer(C_6_H_5_Na_3_O_7_·2H_2_O) and then microwaved for 20 min for antigen retrieval. Endogenous peroxidase activity was blocked with 0.5% (v/v) H_2_O_2_. The slides were then transferred into a humidified chamber, incubated with 5% (v/v) horse serum for 30 min and then incubated with primary antibodies overnight at 4 °C. After primary antibody CD31(Sangon Biothch, D260721,1:200 for IHC), REC8 (Proteintech 10793-1-AP, 1:200 for IHC) incubation, the slides were immersed in peroxidase-labeled secondary antibody for 30 min at room temperature. To detect the antibody-conjugated antigen reaction, the sections were incubated in 3-amino-9-ethylcarbazole substrate-chromogen for 30 min and counterstained with hematoxylin. The evaluation of the staining was performed as described in our pervious study [19]. Quantitative analysis of the immunostained images of human biopsies was performed by positive cell number counting and computerized optical density (OD) measurements with Image Pro Plus 6.0 software (Media Cybernetics, MD, USA).

### Chromatin immunoprecipitation (ChIP) assays

ChIP assays were performed in BGC823 cells by using a SimpleChIP® Plus Enzymatic Chromatin IP Kit as pervious work [20]. Briefly, 1.0 × 10^7^ cells were cross-linked with 1% (w/v) formaldehyde for 10 min and then quenched in 0.125 M glycine for 5 min. Cells were lysed and digested to collect the chromatin. IP was carried out by using the indicated antibody overnight. The precipitated DNAs were analyzed and quantified by using real-time PCR analysis. Primer sequences for *VEGF* (NM_001171622) listed as followed: forward 5′-CGTGTGGAAGGGCTGAGG-3′, reverse 5′-CCGCTACCAGCCGACTTTT-3′,

### Statistical analysis

All statistical analyses and statistical graphing were done using GraphPad Prism 8 software. All the t test was used to determine the significance of differences in the qPCR assay. One-way ANOVA was performed on data from endothelial cell tube formation assay. For correlation analysis, p value of less than 0.05 was considered statistically significant.

## Results

### Downregulation of REC8 in GC was negatively correlated with microvascular density.

**Fig.1:**
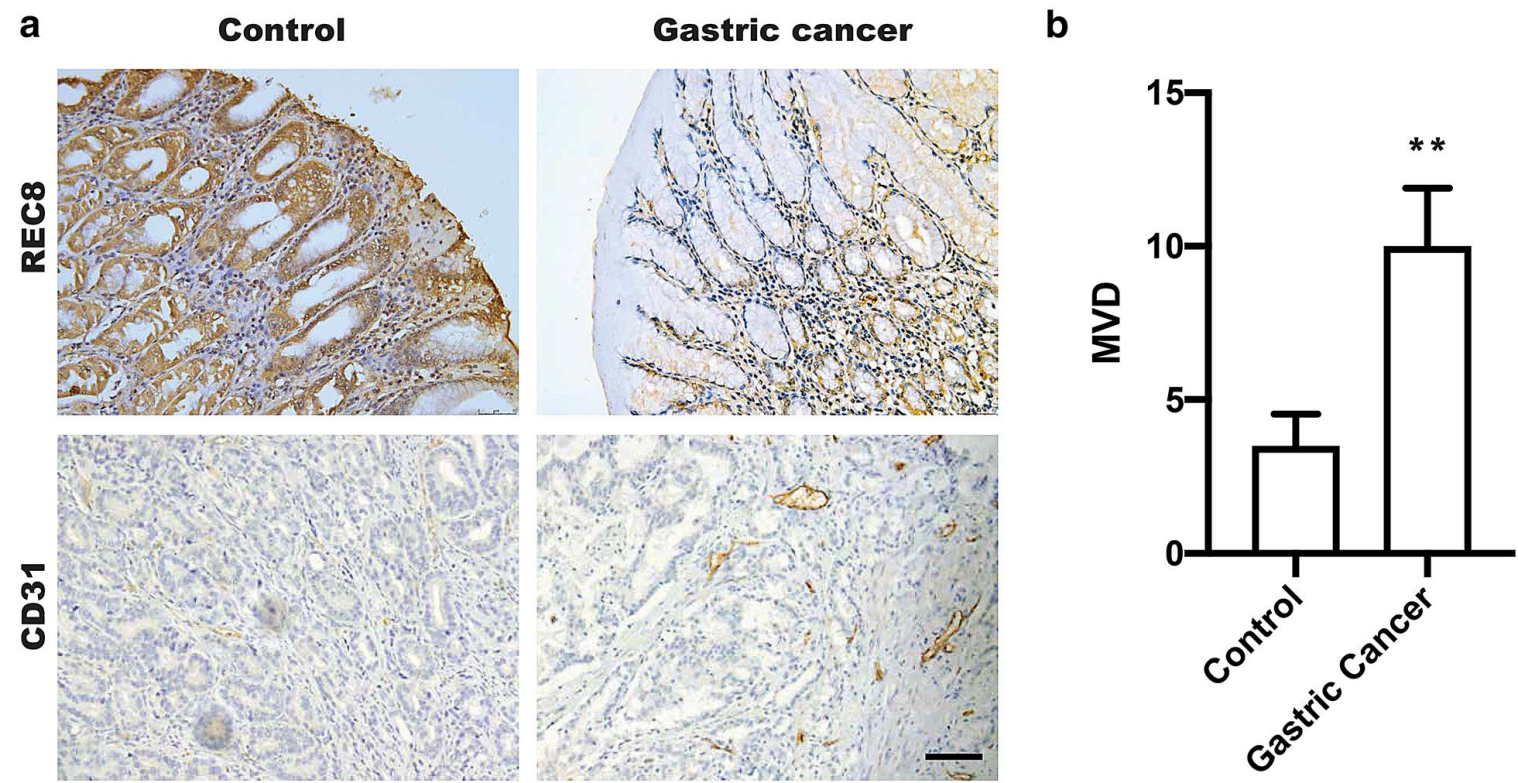
IHC assay showed REC8 expression and MVD in gastric cancer. **a** Representative images of immunohistochemistry staining for REC8 expression and abundance of angiogenesis (CD31 marker) in gastric cancer tissue and paired control. Sar bar = 100 nm; **b** Quantification of MVD in clinical tissue microarrays between cancer tissues (n = 59) and paired controls (n = 16) were analyzed by t-test. **p < 0.01

To further explore the possible clinical relationship between REC8 and microvascular density (MVD), we performed immunohistochemical staining to reveal REC8 expression is extraordinarily reduced in patients with GC (n = 59) compared with that in adjacent tissue (n = 16) (Fig. 1a). Inversely, CD31 (an endothelial cell marker) and MVD were significantly increased in GC compared with adjacent tissue and quantified in Fig. 1b. These results suggested the possible role of REC8 in tumor angiogenesis.

### Inhibition of REC8 expression in gastric cancer cells promoted HUVEC cells tube formation

**Fig.2:**
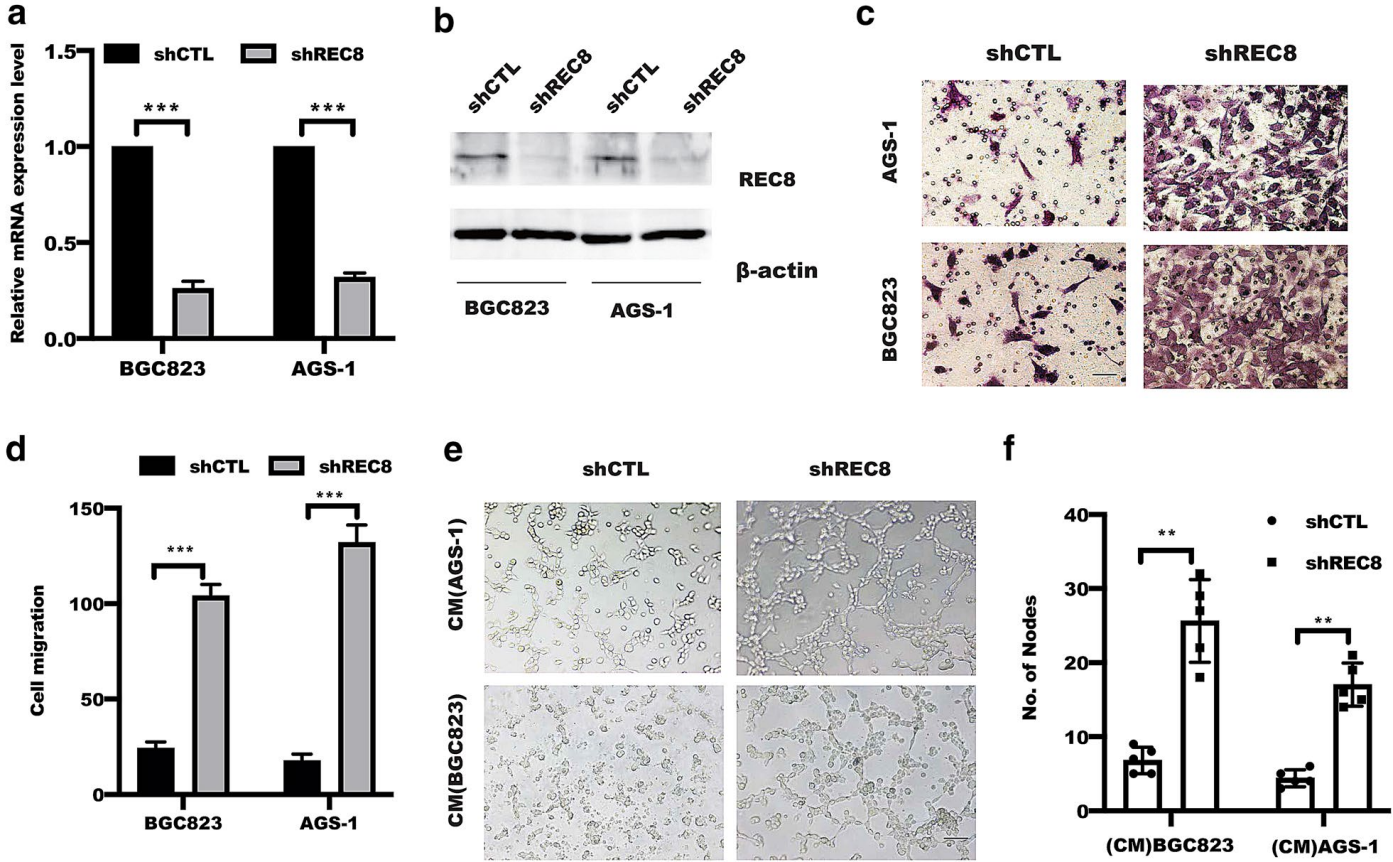
Gastric cancer cells with REC8 depletion promoted chemotactic migration and tube formation of endothelial cells. **a** Real-time and **b** western blotting were performed to examine the REC8 expression at mRNA and protein level. **c**, **d** Conditional medium (CM) from BGC823 or AGS-1 cells infected with shCTL and shREC8 were used to be chemoattractant for HUVECs migration by transwell assays. ***p < 0.001 versus shCTL group by t-tests. Sar bar = 50um; **e**, **f** HUVECs were treated with conditional medium from BGC823 cells treated as indicated. Tube formation of HUVEC cells were visualized by phase contrast inverted microscope (100 ×), **p < 0.01 versus shCTL group by t-tests

To explore possible role of REC8 in the tumor angiogenesis, we established BGC823 and AGS-1 cells that stably expressed REC8 depletion by lentivirus infection and confirmed by real-time PCR and WB (Fig. 2a, b). Next, we investigated the effect of REC8 in gastric cancer cells on HUVECs migration using transwell model system. As shown in Fig. 2c, d, the conditional medium (CM) from REC8-depleted BGC823 or AGS-1 cells significantly increased the chemotactic migration of HUVECs compared with CM from the shCTL group. Further investigation showed that supernatant from ablated of REC8 expression in gastric cancer cells drastically enhanced HUVEC tube formation by a co-cultured with HUVECs (Fig. 2e, f). In summary, these results showed that the critical role of REC8 in mediating tumor angiogenesis.

### REC8 suppressed tube formation in dependent of VEGF

**Fig.3:**
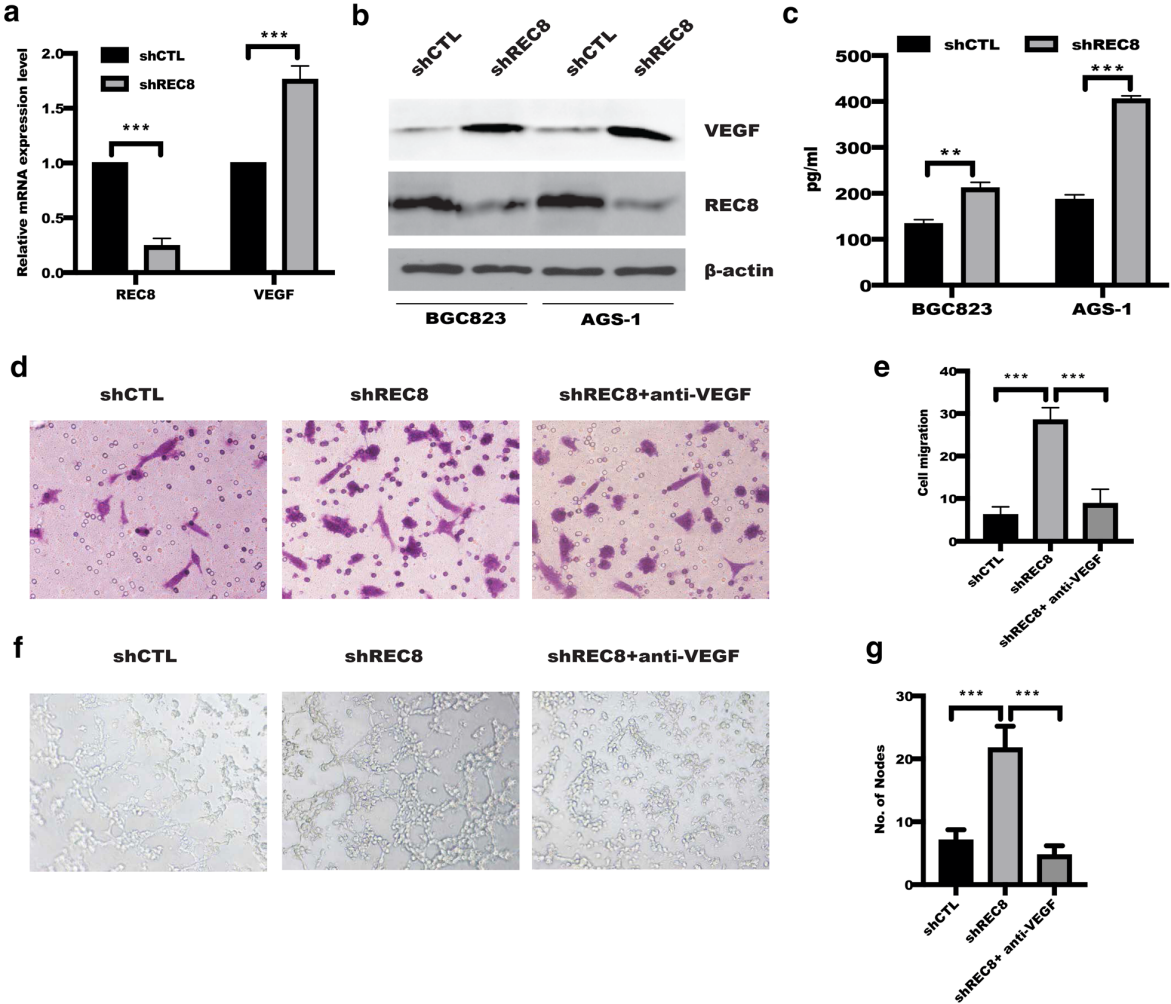
Depletion of REC8 enhanced HUVECs migration and tube formation through upregulation of VEGF in gastric cancer cells. **a**–**c** The mRNA and protein level of VEGF were analyzed by real-time qPCR, western blotting and ELISA in BGC823 and AGS-1 cells. ***p < 0.001, **p < 0.01 verus shCTL. **d**–**g** Transwell assay and tube formation assay were employed to detect HUVEC migration and tube formation with or without neutralizing VEGF antibody in CM from indicated group in BGC823 cells. Quantification was performed to analyze the statistical significance. ***p < 0.001

It is noteworthy that VEGF secreted by cancer cells acted as chemoattractants in promoting motility of endothelial cell during tumor angiogenesis [21, 22], which focused us to seek the potential role of REC8 on VEGF expression. Interestingly, we found that VEGF expression was significantly increased at mRNA level in BGC823 cells with REC8 knockdown (Fig. 3a), in line with this, WB results reveled that depletion of REC8 expression in BGC823 and AGS-1 cells drastically increased VEGF expression level (Fig. 3b), which is also confirmed by Elisa results (Fig. 3c). What’s more, neutralizing VEGF in CM significantly abrogated CM that from REC8-depleted BGC823-induced migration of HUVECs (Fig. 3d, e), leading to inhibit angiogenesis (Fig. 3f, g), implying that REC8 facilitated angiogenesis at least in part by triggering the release of angiogenic factor VEGF. These data indicated that REC8 of gastric cancer cells inhibited tumor angiogenesis through VEGF.

### NF-κB is required for REC8-mediated VEGF expression

**Fig.4:**
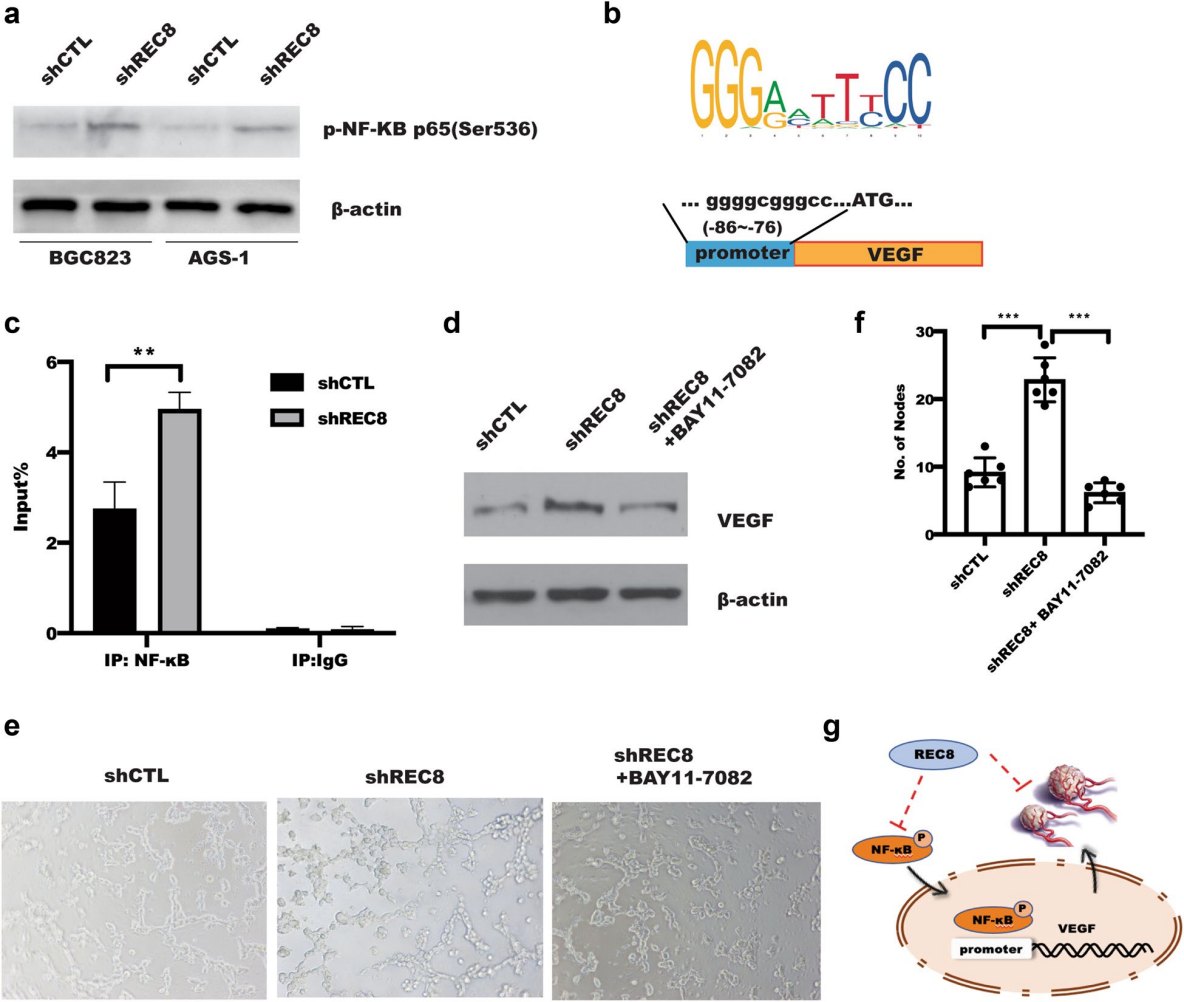
NF-κB is required for VEGF transcription in gastric cancer cell with ablation of REC8. **a** Western blotting was used to detect phosphorylation of NF-κB. **b** A putative binding sequence of NF-κB p65 in VEGF promoter by UCSC analysis. **c** ChIP analysis of the binding of NF-κB to *VEGF* gene promoter in BGC823 cells depleted with REC8. Two-way ANOVA, **p < 0.01, the experiment was repeated three times. Error bars indicate mean ± S.D. **d** Western blotting and **e**–**f** tube formation were applied to measure VEGF in conditional medium from BGC823 cell infected with indicated lentivirus in present with or without NF-κB inhibitor BAY11-7082 treatment. **g** Schematic diagram for the mechanistic role of REC8 in tumor angiogenesis by regulation NF-κB-mediated VEGF in gastric cancer

NF-κB has been reported to play an important role in VEGF expression [23, 24], which focused us to explore the possible effect of REC8 on NF-κB activity. As shown in Fig. 4a, silencing of REC8 expression in BGC823 and AGS-1 of gastric cancer cell promoted the phosphorylation of NF-κB p65 (Ser536), and no significance change of total NF-κB p65 was observed between shCTL and shREC8 (Additional file 1: Figure S1). To further explore the possibility that upregulation of VEGF transactivation resulting from REC8 depletion was caused by increased binding of binding of NF-κB to the *VEGF* promoter sequence, we utilized chromatin immunoprecipitation (ChIP). UCSC online software was used to identify a putative binding sequence, -86 GGGGCGGGCC -76 of NF-κB in the promoter of *VEGF* genes, which is similar to the NF-κB binding consensus sequence GGGRNNYYCC [25] (Fig. 4b). As shown in Fig. 4c, the baseline of the binding of p65 NF-kB to the VEGF promoter region was dramatically increased in response to REC8 depletion. Additionally, inhibition of NF-κB by NF-κB inhibitor BAY11-7082 treatment could reverse the promotion of REC8 knockdown in VEGF expression, leading to attenuate the tube formation (Fig. 4d, e), implying involvement of NF-κB in REC8 tumor biological function.

## Discussion

Up to now, no available reports about the function of REC8 in tumor angiogenesis. In this study, as shown in Fig. 4g, we are for the first time to demonstrate that knockdown of REC8 expression in BGC823 and AGS-1 of gastric cancer cells in vitro significantly contributed HUVECs recruitment through NF-κB mediated VEGF expression, thereby promoting tumor angiogenesis. Downregulation of REC8 was correlated with tumor angiogenesis in gastric cancer tissues. Moreover, slicing REC8 expression significantly promoted HUVECs migration and tube formation through regulating VEGF expression and secretion, Mechanically, REC8 interacted with NF-κB and inhibited NF-κB activity, leading to attenuate NF-κB nuclear translocation. These findings indicated the novel role of REC8 in tumor angiogenesis in gastric cancer and could be alterative therapy strategy to recover REC8 expression and REC8-induced tumor angiogenesis.

REC8, a classically known to be a key component of the meiotic cohesion complex, played a critical role in chromosome dynamics during meiosis, including homology chromosome pairing, crossover recombination, and sister chromatid cohesion during meiosis [11, 26]. It is interesting to find that hypermethylation of the REC8 gene was uniquely more common in follicular thyroid cancer (FTC) and anaplastic thyroid cancer (ATC) [14]. In addition, the study by Yu et al*.* showed that a significant negative correlation between REC8 promoter methylation and mRNA expression by linear regression analysis, suggesting a prominent novel tumor suppressor gene that is epigenetically robustly targeted by the PI3K pathway in thyroid cancer [14]. Moreover, REC8 expression was associated with poor tumor prognosis in patients [27–29], which is not only attributed to cell growth and migration caused by REC8 reduction [30], but also promoted EGR1-mediated EMT [31]. In this study, we further demonstrated the novel function of REC8 in tumor angiogenesis by activation of NF-κB-VEGF-mediated recruitment of HUVECs and tube formation, inhibition of NF-κB by NF-κB inhibitor BAY11-7082 significantly revered the promotion of CM from REC8-depleted gastric cancer cells on tube formation, which would be required to confirm in vivo. What’s more, REC8 interacted with NF-κB, leading to inhibit NF-κB activity. Dynamic change NF-κB p65 activity is critical event in nucleus shuttle [32]. Depletion of REC8 expression in BGC823 cells drastically contributed the binding of NF-κB to VEGF promoter, leading VEGF transcription. However, in addition to VEGF, whether other pro-angiogenic factors, such as IL-6 et al., involved in REC8-mediated angiogenesis, whether REC8 regulated NF-κB activity in classical or non-classical pathway, and whether clinical trials with anti-angiogenic agents suppressed tumor angiogenesis in REC8-dependent manner, these unsolved issues remained to be addressed.

In summary, in addition to the studies showed that the role of REC8 in tumor migration, invasion, cell proliferation, growth and apoptosis in gastric cancer [30]. Our study is the first time to unravel the role and mechanism of REC8 in tumor angiogenesis by regulation of NF-κB-mediated VEGF expression in gastric cancer cells. Further studies are required to elucidate the role of REC8 in tumor immunology, tumor differentiation, tumor metabolism and remodeling tumor microenvironment.

## Conclusion

The current study provided a novel insight into the functional role of REC8 in tumor angiogenesis, suggesting REC8 serves as a negative regulator in tumor angiogenesis through suppressing NF-κB-mediated VEGF, and demonstrating a novel function of REC8 in tumor progression.

## Electronic supplementary material

Below is the link to the electronic supplementary material.

**Additional file 1: Figure S1.** p65 expression in BGC823 and AGS-1 cell treated as indicated. Western blotting was performed to detect the expression of p65 in BGC823 and AGS-1 cell treated with shCTL and shREC8, respectively.

## Data Availability

The datasets generated during and/or analyses during the current study are available from the corresponding author on reasonable request.

## Abbreviations

CM: Conditional medium
ChIP: Chromatin immunoprecipitation
EMT: Epithelial-Mesenchymal Transition
ELISA: Enzyme Linked Immunosorbent Assay
GC: Gastric Cancer
HUVECs: Human Umbilical Vein Endothelial Cells
IL: Interleukin
IP: Immunoprecipitation
IHC: Immunohistochemistry
MVD: Microvascular Density
NF-κB: Nuclear factor-kB
PCR: Polymerase Chain Reaction
UCSC: University of California Santa Cruz
VEGF: Vascular Endothelial Growth Factor
WB: Western blotting
