# Supplementary figures and images for: REC8 suppresses tumor angiogenesis by inhibition of NF-κB-mediated vascular endothelial growth factor expression in gastric cancer cells
Source: Biol Res. 2020 Sep 21;53:41. doi: 10.1186/s40659-020-00307-1 (PMC7507279; doi:10.1186/s40659-020-00307-1)

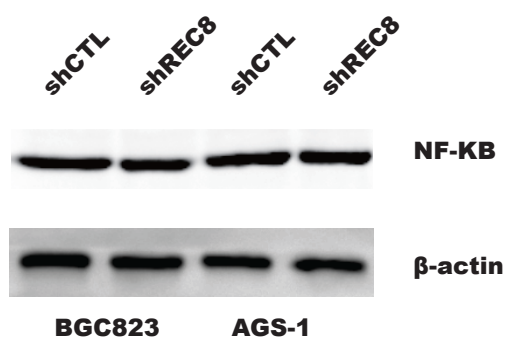

Supplement: Supplementary file 1 — Additional file 1: Figure S1. p65 expression in BGC823 and AGS-1 cell treated as indicated. Western blotting was performed to detect the expression of p65 in BGC823 and AGS-1 cell treated with shCTL and shREC8, respectively. [file 40659_2020_307_MOESM1_ESM.pdf]
